# An Historical View of Surgery in the Sixteenth
Century

**Published:** 1799-12

**Authors:** 


### *An Historical View of Surgery in the Sixteenth Century.*

[ Concluded from p. 364—368 of our last Number. ]

THE obstetric art, this important branch of surgery, began to emerge from its barbarity during the sixteenth century, and to excite the attention of surgeons more than it had hitherto done. There appeared several introductions to midwifery, the greater number of which, however, contained much useless and abstruse reasoning on the generation of man, and the vitality of the embryo in certain months, while they were extremely deficient in well-founded and practical rules for facilitating delivery.

Most of the writers of that century followed the principles of EUCHARIUS RÖSSLIN, sometimes called RHODION; who wrote, in the German language, a work printed at Augsburg, in 1551, 8vo. entitled, "*The Garden of Roses for Pregnant Ladies and Midwives.*" His representations of the preternatural situation of the child; his proposals for accelerating the expulsion of the fœtus by emollient external applications, and stimulants administered internally; his advice to pay constant attention, that the child's head may be protruded first; and lastly, his rule that the dead child should be extracted with hooks, knives, and other dangerous instruments;—all these particulars have been repeated by the obstetric writers of that age, without any essential improvement. Thus, VALLERIOLA* praises the surgeons in Provence, who handled these destructive weapons with great skill and ability.

JASON A PRATIS† also wrote a Treatise on Midwifery, which abounds in whimsical and absurd notions; insomuch, that it scarcely contains one original or sensible idea. Not less inconsistent is the Treatise entitled, "*The Book for Midwives,*" written in German, by WALTER HERMAN RYFF, printed at Frankfort, 8vo. 1569. JACOB RUEFF, a surgeon at Zürich, on the contrary, is the author of a work, which, besides the principles of the Arabian writers borrowed from RÖSSLIN, contains a delineation of the first forceps, which, however, can be applied only to dead children, for the compression of the head.‡ RUEFF likewise

* *Observ. Medic.* lib. v. c. 2. p. 319.

† *De pariente et partu,* 12mo. Amsteld, 1657.

‡ *De conceptu & generatione hominis,* f. 30. 4to. Tigur, 1554.

likewiſe treats, with conſiderable accuracy, on the cauſes to which the retention of the ſecundines* is to be aſcribed. In the works of MERCURIALIS† and PARE,‡ we meet with ſimilar principles and precepts. The latter of theſe writers is particularly apprehenſive of the dangerous conſequences ariſing from the retention of the placenta.§

§. 20. JACOB GUILLEMEAU excels all the writers hitherto mentioned, both in fundamental erudition and rational principles, tending to facilitate the delivery of parturient women. Although he ſtill propoſes the old faſhioned inſtruments for dilating the vagina, and is not ſufficiently acquainted with the turnings of the child, as he in many caſes prefers the delivery by the feet to natural birth; yet his animadverſions on the ſurgeons, for neglecting the ſtudy ot midwifery,|| are truly laudable; and his method of effecting the *accouchement forcé*, in flooding caſes, has been renewed and confirmed by the works of later writers on that ſubject.¶ He alſo ſeriouſly diſſuades the practitioner from attempting to promote the diſcharge of the after-birth, by violent means.**

HIERONIMUS MERCURIALIS, of Rome, likewiſe deſerves to be included among the better claſs of chirurgical writers, though he is ſtill attached to ſuperſtitious notions and eſtabliſhed prejudices. He was a pupil of ARANZI, but had embraced the religious order of predicants, and at the ſame time practiſed ſurgery at Milan: yet, as this profeſſion was conſidered as incompatible with the dignity of a religious order, he abandoned the monaſtery, and travelling through many cities of Italy, he exerciſed his favourite art without moleſtation from the monks. His longeſt reſidence was at Peſchiera and Civita Vecchia: and after having viſited France and Spain, he at length returned to the fraternity of his order, at an advanced age. His book, which he publiſhed under the name of SCIPIO MERCURIO, has been tranſlated into moſt languages, and chiefly contains a collection of the principles and obſervations

* *De conceptu & generatione hominis*, lib. iii. c. 4. f. 25. a.

† *De morb. mulier.* lib. ii. c. 2. p. 49; in BAUHIN. *Gynaec.* vol. ii.

‡ *Oeuvres d'Ambr. Paré.* liv. xxiv. ch. 33. p. 608.

§ *Ibid.* ch. 18. p. 602.

|| *Guillemeau, de la groſſeſſe et de l'accouchem. des femmes*, p. 258.—Oeuvres, fol. Paris, 1598.

¶ *Rigby von Mutter-Blutflüſſen.* 8vo. Leipzig. 1786.

** *Guillemeau*, p. 280.

obſervations of his predeceſſors. He differs, however, from them in many eſſential points: thus, he cenſures Rueff, for preferring the delivery by the feet to that in the natural way.* The head uſually preſents itſelf in ſuch a manner, that the face is behind, but frequently the contrary takes place, and even then, it is not a preternatural caſe.† He endeavours to prove, by a very curious and whimſical calculation, that children born in the eighth month, cannot live;‡ a ſuperſtitious notion entertained by the antients.—The ſituation in which he places the mother, in preternatural births, is truly ſhocking; for he cauſes a number of pillows to be placed under her back, and piled up ſo high, that the head entirely declines,, and the legs likewiſe are placed in a recumbent poſture.

§ 21. As Mercurialis forcibly recommends the *Cæſarean ſection*, and has been an eye witneſs to a ſucceſsful caſe in that operation, the hiſtorian is naturally led to inveſtigate its antiquity. Prof. Sprengel obſerves, that he has endeavoured to ſhew, in his former diſquiſitions relative to this ſubject,|| that Nature has probably pointed out to mankind the neceſſity of this operation; as there are ſeveral caſes on record, where, in a *partus extra-uterinus*, an external ulcer appeared on the abdomen, and thus the dead body of the child was diſcharged. In the ſequel of theſe pages, we ſhall have occaſion to mention ſome intereſting caſes of this nature, from the writers of the ſixteenth century.

It may farther be eaſily conceived, that this operation was undertaken in the earlieſt times, from a natural love and ſympathy for a living being, eſpecially if the pregnant mother had recently died: and thus, the firſt traces of this practice are, perhaps, to be found in fabulous hiſtory. The Greeks relate, that Jupiter, when on a viſit to Semele, one of the daughters of Cadmus, having brought down his thunder-bolts from Olympus,

---

* *Mercurii la commare ó raccoglitrice*, lib. ii. c. 5. p. 120.—4to. Verona, 1662.

† *Ibid.* p. 26.

‡ *Ibid.* lib. i. c. 8. p. 39. "The fœtus," ſays he, "is formed either in 35 or 45 days. Its formation, therefore, is always imperfect, when accompliſhed in forty days. The double number of days requiſite to form the fœtus, points out the time of motion; and this, if multiplied with 3, gives the time of birth. Thus is 40+2=80, and 80+3=240= eight months. *Ergo.*

§ The profeſſor here refers the reader to his elaborate *Treatiſe on the Cæſarean ſection*, inſerted in Pyl's *Repertory of Medical and Political Juriſprudence*, (in German,) part or vol. ii. numb. i. p. 116. and ſeq.

Olympus, he handled them with so little circumspection, that a flash of lightning struck the house of Semele, and burnt it together with her person. In great haste, on the spur of this emergency, Jupiter commanded Hermes to rescue the imperfect fœtus, then seven months old, by cutting it from the burning body of Semele. Jove concealed the child in his hip for nearly three months, and afterward produced Dionysos or Bacchus*. The Roman mythology informs us, with respect to Æsculapius, that his father, Apollo, had cut him out of the womb of his mother, Coronis, when she was placed on the funeral pile;† and that Æneas combated one of his adversaries, named Lychas, who had been born in a similar manner, and, on that account, was sacred to Apollo.‡

The Cæsarean section must have been successfully performed at a very early period of time; as Numa Pompilius enacted the *lex regia*,§ that no pregnant woman should be buried before the fœtus had been extracted by dissection. Conformably to this law, PLINY || relates, that Claudius, the first of the Cæsars, and a certain Cæso, of the family of Fabius, had thus been given to the world; and that from this remarkable circumstance they had received their names. We are farther told, that MANILLUS, who conquered Carthage in the third Punic war, and SCIPIO AFRICANUS, came into the world in a similar manner.¶ The same royal law has been repeatedly enforced by the Church of Rome; a fact for which the authority here quoted, will be deemed sufficient evidence.**

§ 22. We find by the first instance on historical record, that the Cæsarean operation had been performed on a living subject, in the beginning of the sixteenth century. It is, however, to be regretted,

---

* *Lucian. Dialog. Neptun. et Mercur.* p. 202. Opp. vol. i. ed. *Graev.* 8vo. Amstel. 1687.

† *Ovid. Metamorph.* lib. ii. fab. ix. v. 680.

‡ *Virgil. Æneid.* lib. x v. 315. "Inde Lycham ferit, exsectum jam matre peremtâ

"Et tibi Phœbe sacrum."

§ *Digest.* lib. xi. tit. 8. *De mortuo inferendo*, l. 2. "Mulier quæ prægnans mortua, "ne humator antequam partus ei excidatur, quei secus faxit spei animantis cum gra- "vida occisæ reus estod."

|| *Plin. Hist. Natur.* lib. vii. c. 9.

¶ *Harduin. not. et emendat. ad Plin.* p. 432.

** *Martene et Durande* Collect. amplifs. vol. vii. p. 1282.

gretted, that moſt of the chirurgical operations have been firſt attempted by ignorant men: ſuch was Nufer,* of Turgau, by profeſſion a ſow-gelder, who had the reſolution to undertake that dangerous ſection on the body of his wife, with the happieſt ſucceſs. It is farther aſſerted, that the illuſtrious Andrew Doria was born by the aid of the Cæſarean ſection.†

Towards the middle of that century, a remarkable caſe happened at Vienna, which obviouſly proved that, in extra-uterine conception, or if the fœtus by a rupture of the womb enters the abdomen, Nature herſelf appears to point out the Cæſarean operation. The following is a ſhort hiſtory of the caſe: The wife of Wolczer, an innkeeper, after having been delivered of ſeveral children, became pregnant in 1545. When the time of childbirth approached, and the pains became violent, ſhe made great exertions. On a ſudden, a report was heard, as if ſomething had burſt within her body, and the milk flowed to the breaſt. The pains from that time ceaſed, but the child did not appear, and the abdomen increaſed in ſize. She became cachetic, and had a fetid diſcharge from the vagina: at length, in 1548, an abſceſs took place in the abdomen, from which a ſimilar ichor, and, in 1549, even a bone was evacuated. As the patient grew progreſſively worſe, ſurgeons and phyſicians were conſulted. Matth. Cornax, Prof. at Vienna,‡ followed the parental hints of Nature, enlarged the orifice of the abſceſs, and ſucceſsfully extracted the child, in a ſemi-putrid ſtate! The mother, after this period, ſo completely recovered that within the next two years ſhe again became pregnant. When the time of her delivery approached, the child, upon examination, was found to be in full vigour, but the natural paſſages were remarkably narrow; while the cicatrix of the old wound was moiſt and apparently opening of itſelf. Cornax propoſed to open it by inciſion; but the woman's mother oppoſed this rational ſtep. The judicious practitioner was conſequently obliged to leave the unfortunate patient without a proſpect of relief, and ſhe expired in a very ſhort time. After her demiſe, the old wound was opened, and the child extracted, which appeared to have but recently died.§

* *Bauhin in Append. ad Rouſſeti hyſterotomot.* p. 37.

† *Venoſta* diſcorſo interno alla generazione e naſcimento degli uomini, p. 47. 8vo. Venez. 1562.

‡ *Eloy*, vol. i. p. 711.

§ *Dodon.* exempl. medic. obſerv. p. 306 et ſeq.—*Marcell. Donat.* lib. iv. c. 22. f. 239. —Diom. *Cornar.* hiſtor. admir. 6. p. 13.

Similar cases were, in that age, observed by ÆGID. HERTOGE, of Brussels,* and ACHILLES PIRMINIUS GASSARUS, a very learned Physician at Augsburg.† FIORAVANTI, an Italian charlatan, also relates a case in which the Cæsarean section was performed with tolerable success, so that the patient was only troubled afterwards with a *prolapsus uteri et vesicæ.*‡ The celebrated PARE was likewise acquainted with several successful cases of this kind, yet he does not recommend the operation forcibly; as the life of the mother is always in danger.§ The earliest scientific treatise on this subject, illustrated by figures, we find in CHARLES ETIENNE; || and FELIX PLATER quotes a remarkable case in which a dead fœtus was extracted by section from the body of a living mother.¶ Several other instances of a similar nature were also collected by MORITZ CORDÆUS.**

§ 23. The Cæsarean operation excited the greatest attention among medical men, when FRANC. ROUSSET, surgeon to the Duke of Savoy, wrote a masterly performance in its vindication. He first appeals to experience, respecting the success of the operation observed partly by others, and partly by himself. The history of the first case he relates, is by far the most curious and memorable ever recorded. A woman, near Milly, was SIX TIMES successfully delivered by the Cæsarean section, but died with her seventh child; as the surgeon who had formerly performed that operation, was no more.†† ROUSSET next endeavours to prove from analogy, that wounds of the abdominal muscles, and the peritonæum, are not more dangerous than injuries of the uterus itself: ‡‡ he farther maintains, that in cases of mal-conformation of the pelvis, too large a size of the child, or extra-uterine conception, there remain no other means of accomplishing delivery than the Cæsarean operation. ||||

The assertion of SUE, the younger, in his superficial History of Midwifery, that ROUSSET has borrowed his remarks from PLATER, is groundless;

* *Dodon.* exempl. medic. observ. p. 321.

† *Ibid.* p. 328.—Vid. his Biography in *Adami*, p. 233.

‡ Tesoro della vita umana, p. 170. 8vo. Venez. 1570.

§ Liv. xxiv. ch. 33, p. 608.

|| De dissect. part. corp. human. Lib. iii. cap. 1, p. 261. fol. Paris, 1546.

¶ Observat. med. lib. i. p. 212.

** Commentar. in *Hippocrat.* libr. de morb. mul. lib. ii. p. 250.

†† *Hysterotomotokia*, S. I. c. 5, p. 504.—In *Bauhin's Gynaec.* vol. ii.

‡‡ *Ibid.* S. II. p. 511.

|||| *Ibid.* S. I. c. 3, p. 502.—S. II. p. 535.

lefs; as this writer publifhed his obfervations two years after the work of ROUSSET. In 1582, BAUHIN publifhed a Latin tranflation of the laft mentioned work, corroborated the opinion of ROUSSET, and was fo warm an advocate for the Cæfarean operation, that after this period it was performed by various furgeons in France. But, as they did not always proceed upon an accurate diagnofis, they confequently did not meet with uniform fuccefs. A work written by GUILLEMEAU,* in which five fatal cafes of the Cæfarean fection are recorded, induced ROUSSET to publifh a defence of that operation: † here he treats the fubject with fuch acutenefs and ingenuity, as cannot fail to infure the approbation of the reader. Soon after this, JAC. MARCHAND compelled him, by an undeferved libel,‡ to anfwer the objections of his adverfaries a fecond time, and with much feverity.§ We are affured by Mercurialis,|| that this operation was then very common in France, though his veracity is queftionable, from the extravagance of his expreffions. It was alfo introduced into Italy by JUL. CÆS. ARANZI, who was a fuccefsful operator:¶ —CORN. GEMMA,** and HOR. AUGENIUS,†† relate fimilar inftances of operations which had a fortunate iffue in that country.

* De la groffeffe et de l'accouchement des femmes, p. 190.

† *Rouffeti* affertio hiftorica et dialog. apologeticus pro cæfareo partu, 8vo. Paris 1590.

‡ *Marchand* in *Rouffeti* apologiam declamatio, 8vo. Paris, 1598.

§ *Rouffeti* brevis apologia pro partu cæfareo in dicacis cujufdum chirurguli theatralem invectivam, 8vo. Paris, 1598.

|| *Mercurii* la commare ò raccoglitrice, Lib. II. c. 28, p. 169.—"The Cæfarean fection," fays he, "is as common in France, as venæfection for the head-ach is in Italy."

¶ *Craton.* Epift. Lib. V. p. 297.

** Cyclognom. Lib. II. c. 6. p. 74.

†† Epiftol. Lib. V. 2. p. 379.

